# A Diagnostic Dilemma and Classification Conundrum: Atypical Histiocytic Neoplasm Presenting as a Calvarial Mass

**DOI:** 10.7759/cureus.54828

**Published:** 2024-02-24

**Authors:** Shabbir Haiderbhai, Leesha Heitkamp, Austin Nickell, Ellen Erie, Laura Nichols

**Affiliations:** 1 Internal Medicine, Sanford Health, Fargo, USA; 2 Internal Medicine, University of North Dakota School of Medicine and Health Sciences, Fargo, USA

**Keywords:** indeterminate cell histiocytosis, atypical presentation, skull mass, non-langerhans histiocytosis, langerhans cell histiocytosis(lch)

## Abstract

Histiocytic disorders are a wide range of disorders arising from abnormal proliferation and infiltration of dendritic cells. The Histiocyte Society has arranged the disorders into five main groups: L, C, M, R, and H. We present a case in which an elderly woman presented with a solitary osseous lesion in her skull in the right anterior calvarium. Biopsy and histological studies were strongly positive for cyclin D1; positive for CD68, S100, and ZBTB46; weakly positive for OCT2; and equivocal for ALK1 and CD163. Genomic studies also identified KRAS and GPS2 mutations. KRAS-positive genomic analysis favors a diagnosis of histiocytoma, while the solitary calvarium and spontaneous resolution with remission favor a diagnosis of Langerhans cell histiocytosis (LHC). Despite the strong clinical evidence favoring LCH, our patient’s clinical and histologic features did not fit any of the five histiocytic categories and were classified as an atypical histiocytic disorder.

## Introduction

Histiocytic disorders are a wide range of disorders that affect both children and adults, predominantly the former [1,2]. In 2016, the Histiocyte Society published an updated classification system and arranged the disorders into five main groups: the L group, which includes both Langerhans cell histiocytosis (LCH) and Erdheim-Chester disease (ECD); the C group, which includes cutaneous and mucocutaneous non-LCH; the M group, which includes both primary and secondary malignant histiocytoses; the R group, which includes Rosai-Dorfman disease (RDD) and other non-C/L/M/H histiocytosis; and the H group, which includes hemophagocytic lymphohistiocytosis (HLH) and macrophage activation syndrome (MAS) [1]. Diseases within each group are distinct, with over a hundred different histiocytic disorders identified to date. Each presents with different symptoms and has different diagnostic features and distinct management algorithms [1]. Recent National Comprehensive Cancer Network (NCCN) guidelines include the diagnosis and treatment of the three most common histiocytic disorders: LCH, ECD, and RDD [2].

LCH is the most common histiocytic disorder, characterized by abnormal proliferation and infiltration of dendritic cells (histiocytes). *Histiocytes* has historically been a term used to describe tissue cells. In the context of LCH, it refers to dendritic cells, mononuclear cells with phagocytic features. The clonal proliferation of the aberrant myeloid precursor cells that eventually differentiate into dendritic cells is the underlying pathophysiology of this disease [3].

It has been hypothesized that the proto-oncogene BRAF V600E mutation, a central aspect of the mitogen-activated protein kinase (MAPK) pathway, is a key driver of this disorder. It has been found that the BRAF mutation is found in half of childhood LCH cases and less than half of adult cases [4]. 

The reported incidence of LCH is more prevalent in children, with a reported two to nine cases worldwide per million every year, with a median age of diagnosis of three years old [5]. Adult cases of LCH are even rarer, with one to two cases per million reported every year. Most of these adult cases of LCH are usually disseminated diseases [6].

Symptomology of LCH differs based on the organ system affected and varies widely based on each case and presentation. Commonly involved organs include bone, skin, lungs, pituitary, central nervous system, and lymph nodes. LCH can present with just one system involvement, but as described previously, most presentations of LCH are disseminated diseases involving multiple systems [7]. Usual presentations of adult-onset LCH include a skin rash, dyspnea, lower urinary tract symptoms, weight loss, fever, bone pain, ataxia, and memory problems. In cases involving organ symptoms, bone is the most commonly affected site, along with skin and pulmonary involvement [7].

LCH is difficult to diagnose not just because of its rarity, but involvement of multiple organ systems and the overlap of symptoms with other disorders. LCH should be distinguished from other histiocytic disorders based on the histiocytic classification systems, which include but are not limited to, Langerhans cell sarcoma (LCS), ECD, RDD, and HLH [1].

The breadth of diseases and pathology seen in histiocytic disorders makes the classification of such diseases a complex and difficult process. These disorders can present similarly both clinically and radiologically as primary central nervous system tumors but rarely do truly involve the CNS, providing an additional layer of involvement as a mimic [8]. Herein, we present a case of a woman who presented with a single calvarial lesion that did not fall under any of the aforementioned histiocytic categories, thus demonstrating the complexities associated with both the classification and management of such patients.

## Case presentation

A 77-year-old female with a past medical history significant for type I diabetes mellitus with long-term insulin use presented to dermatology with concerns about a right-sided head lump. The patient reported the lesion had recently grown and noted an additional tender lesion. A physical exam at the time demonstrated two subcutaneous nodular masses on the right anterior parietal scalp, with initial concern for a pilar cyst. The patient was referred to general surgery for additional evaluation and observation was recommended with consideration for imaging with progression. 

**Figure 1:**
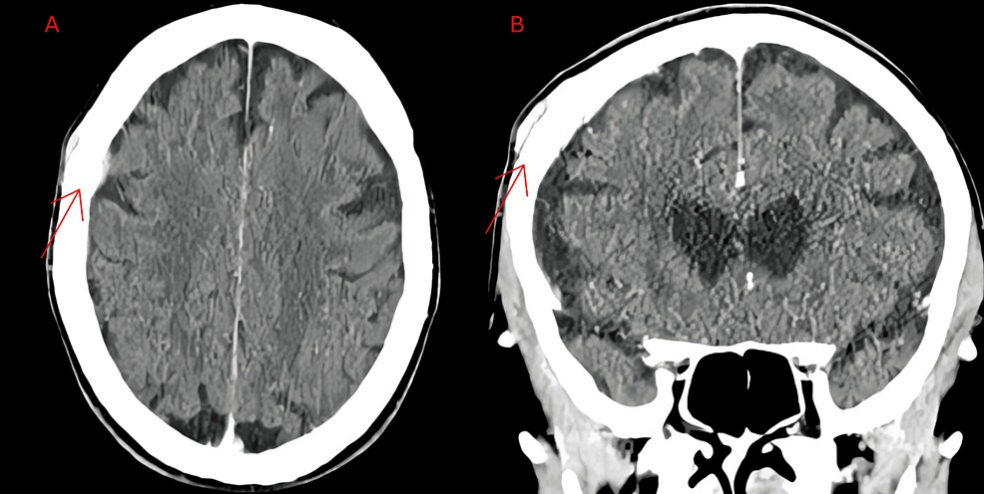
Computed tomography of the head showing an intracranial dural-based enhancing mass (red arrow) measuring 2.8 cm, which appears to extend through frontoparietal calvarium with a scalp component of similar size: (A) axial and (B) coronal views.

Shortly thereafter, the patient reported changes in the lump, including an increase in the size of the lump, frequency of associated headaches, sinus issues, and allergies. Diagnostic studies included a CT head with contrast, revealing an intracranial dural-based enhancing mass measuring 2.8 cm. It appeared to extend through the frontoparietal calvarium, with a scalp component of similar size. There was also a radiographic suggestion of extension into the bone based on mottled bone appearance (Figure 1). Laboratory studies showed a complete blood count (CBC), creatinine levels, and serum protein electrophoresis (SPEP) that were within normal limits. C-reactive protein (CRP) was within the normal range as well, and erythrocyte sedimentation rate (ESR) was mildly elevated at 40 mm/hour (reference range of 0-29 mm/hour).

**Figure 2:**
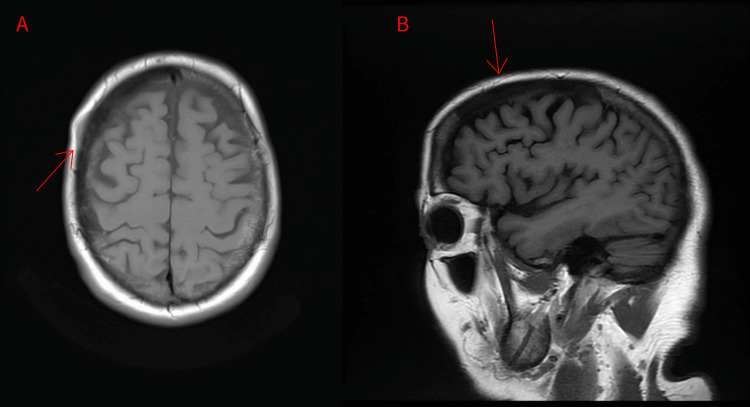
Magnetic resonance imaging showing T1 hypointensity involving the right anterior calvarium (white line) measuring 8.5 cm from the (A) axial and (B) sagittal views. There is plaque-like enhancement along the inner and outer tables associated with this lesion.

Magnetic resonance imaging (MRI) of the brain performed for additional characterization showed a T1 hypointensity involving the right anterior calvarium measuring 8.5 cm in the anteroposterior (AP) dimension. There was plaque-like enhancement along the inner and outer tables associated with this lesion without subjacent brain edema. Findings favored right frontoparietal calvarial meningioma or marrow replacing lesion with plaque-like enhancement along the inner and outer tables (Figure 2).

The patient was subsequently referred for neurosurgical evaluation. Evaluation by neurology and neurosurgery was completed and differential continued to include meningioma, marrow replacing lesion, or less likely a metastatic disease process. A repeat MRI three months after demonstrated a slight interval increase of the right frontoparietal calvarium of about 11 cm, with *epidural and superficial soft tissue extension*. She returned for a follow-up with neurology and neurosurgery. Given the progression between images, a CT-guided biopsy was performed. Cytology reported *atypical histiocytic infiltrate* based on findings of fibrous tissue infiltrated by a spindled population of atypical histiocytes. The cells had small irregular nuclear contours with inconspicuous nucleoli and abundant pink cytoplasm. Immunoperoxidase stains were conducted, revealing the following results: strongly positive for cyclin D1; positive for CD68, S100, and ZBTB46; weakly positive for OCT2; and equivocal for ALK1 and CD163. The stains were negative for CD1a, CD3, CD20, CD21, CD30, CD35, AFB, BRAF V600E, EMA, Factor XIIIa, GMS, langerin, and SOX10.

Genomic profiling was completed using xT Targeted Panel testing by Tempus Labs, Inc., Chicago, IL, which is a custom oncology testing panel used to evaluate 648 genes with single nucleotide variants. Genomic variants identified included KRAS (pG13D-GOF, missense variant of exon 2 with variant allele fraction 16.7%) and GPS2 (p.E46fs-LOF, frameshift-LOF with variant allele fraction 23.2%).

To assist with the delineation of diagnosis, extensive laboratory evaluation was done. CBC and comprehensive metabolic panel were grossly within normal limits, albeit mildly decreased estimated glomerular filtration rate (eGFR) and urine osmolality attributed to dehydration. Otherwise, hormonal and neuroendocrine markers were all within reference range (Table 1). A fluorodeoxyglucose (FDG) positron emission tomography (PET) was completed, which showed FDG avid mass centered in the right frontal bone suspicious of histiocytic neoplasm, mildly enlarged FDG avid right preauricular lymph node suspicious of nodal involvement and elevated FDG uptake within left glenoid, left second rib, and L3 vertebral body.

**Table 1:** Laboratory evaluation completed in conjunction with biopsy and cytology studies. eGFR, estimated glomerular filtration rate; LDH, lactate dehydrogenase; CRP, C-reactive protein; ESR, erythrocyte sedimentation rate; FSH, follicle-stimulating hormone; LH, luteinizing hormone; ACTH, adrenocorticotropic hormone; TSH, thyroid-stimulating hormone; IGF-1, insulin-like growth factor-1

Tests completed	Results	Reference range
eGFR	55 mL/min/1.73 m^2^	>90 mL/min/1.73 m^2^
Serum glucose	147 mg/dL	70-100 mg/dL
LDH	167 U/L	140-280 U/L
CRP	<3.0 mg/L	<3.0 mg/dL
ESR	22 mm/hour	0-20 mm/hour
Urine Osm	199 mOsm/kg	500-850 mOsm/kg
Serum Osm	289 mOsm/kg	275-298 mOsm/kg
FSH	65.9 IU/L	25.8-134.8 IU/L
LH	39.8 IU/L	14.2-52.3 IU/L
Estradiol	14 pg/mL	10-50 pg/mL
Prolactin	6.2 ng/mL	<25 ng/mL
Morning ACTH	18 pg/mL	9-52 pg/mL
Morning cortisol	11 mcg/dL	5-25 mcg/dL
TSH	2.9 mIU/L	0.5-5 mIU/L
IGF-1	89 ng/mL	50-160 ng/mL
HgbA1c	7.3 %	<7%

She was later evaluated by hematology with notable changes in symptoms, including fatigue and cognitive slowing, balance impairments, lower extremity weakness, visual changes, dysphagia to solids, and nausea. Based on her previous evaluations and testing, she was diagnosed with histiocytic neoplasm; however, the specific subtype was unable to be determined as the immunoperoxidase stains, labs, and imaging were not conclusive to a specific, typical subtype of histiocytic neoplasm. Indications for treatment were discussed, including observation, chemotherapy, or targeted drugs such as mitogen-activated extracellular pathway (MEK) inhibitors. Given that she was minimally symptomatic and had no involvement of the central nervous system (CNS) and opted for observation with future repeat positron emission tomography-CT (PET-CT). In the interval, her headache and visual changes seemingly improved as there was noted regression in the skull lesion.

## Discussion

This patient presented with an 8.5 cm hypointense right intracranial mass that extended through the frontoparietal calvarium with scalp involvement. This lesion favored a benign meningioma due to its dural-based presentation, and thus, observation was elected. Repeat imaging two months later showed further growth of the lesion up to 11 cm, with new extraosseous involvement. Extracranial extension of an intracranial meningioma is unusual [2], making a benign meningioma less likely and warranting CT-guided biopsy and immunohistochemical (IHC) staining. The findings of the CT-guided biopsy, IHC staining, and Tempus genomic profiling were suspicious for a histiocytic process.

While there are over 100 subtypes of histiocytosis, the Histiocyte Society has classified these subtypes into five main disease groupings: (1) Langerhans-related (L group); (2) cutaneous and mucocutaneous (C group); (3) malignant histiocytosis (M group); (4) RDD (R group); (5) HLH and MAS (H group) [1].

ECD is an L-group histiocytic disease that was initially considered as a differential, given the positive IHC staining for CD68, S100, and CD163 (equivocal) and negative staining for CD1A and langerin (CD207) [2]. Bone involvement affects almost all patients with ECD, although CNS involvement in the form of dural or parenchymal infiltrates is rare [9]. When parenchymal infiltrates are found, the most frequent CNS findings include endocrine disorders such as diabetes insipidus (25%-50% of patients) [2,9]. Although this patient did have an extra-axial mass involving the dura, no other CNS endocrine findings were reported. Additionally, the lack of retroperitoneal infiltration with periaortic fibrosis, renal failure, retroperitoneal infiltration, urethral obstruction, and exophthalmos make this diagnosis less likely [2,9]. 

It is critical to consider another L-group disorder termed LCH, the most prevalent histiocytic disorder [10]. It presents with incidental osseous lesions in 78% of cases, as observed in our patient [11]. Furthermore, our patient’s spontaneous improvement in the skull lesion without systemic therapy also favors LCH. However, the absence of the classic rash, diabetes insipidus, as well as negative CD1A and langerin (CD207) staining makes the diagnosis unlikely. The osseous involvement observed on MRI goes against the characteristics of C-group histiocytic disorders, which are defined by their localization to the skin and/or mucosa [1,2].

The strong cyclin D1 staining also raises the possibility of a secondary malignant histiocytosis (M group). These histiocytoses, however, often arise simultaneously with other hematologic lymphoproliferations, including follicular lymphomas, hairy cell leukemia, chronic lymphoblastic leukemia, or acute lymphoblastic leukemia [1,2]. Our patient had no past medical history of hematologic cancers, and further workup at the time of diagnosis was unrevealing, making this diagnosis less likely. The cyclin D1 staining pattern also increased the likelihood of R-group histiocytic disease, but the absence of substantial bulky lymphadenopathy, classic B symptoms, and plasmacytic infiltrate on the biopsy specimen made this diagnosis less likely [1,2,12]. Finally, the patient did not have clinical symptoms or laboratory findings suggestive of the Histiocyte Society’s H group, including fever, cytopenias, splenomegaly, hypertriglyceridemia, or elevated ferritin [2].

Although treatment for histiocytic disorders varies widely based on system involvement, the 2019 annual meeting of the Histiocyte Society found international consensus for the treatment of LCH. Single osseous lesions are usually observed alone or treated with curettage for biopsy purposes. If the lesions recur, radiation therapy can be considered. For adults with multiple osseous lesions or multisystem involvement excluding the CNS or at-risk organs, systemic therapy with cytarabine and cladribine should be considered alongside surgical evaluation for unstable bone lesions and further radiation therapy. For patients with multisystem involvement, including CNS and at-risk agents, VRAF B600E mutation-positive LCH should be treated with the respective BRAF inhibitor such as vemurafenib. For individuals without this specific mutation, the current recommendations include systemic therapy with cytarabine or cladribine or consideration for chemotherapy [5].

Recent studies have also shown great efficacy for cobimetinib, a mitogen-activated extracellular pathway (MEK) inhibitor. This pharmaceutical agent has shown great effect in all histiocytic neoplasms, regardless of genotype. Of the 18 patients studied, 89% of patients showed an overall response rate as evidenced by PET testing [13].

Ultimately, the patient was diagnosed with atypical histiocytosis as her clinical presentation and laboratory analysis did not fit into the five disease groupings recognized by the Histiocyte Society. However, the KRAS mutation points toward a histiocytoma. Her predominantly bony presentation and spontaneous remission may favor LCH despite a contradictory IHC staining profile [1,2].

## Conclusions

Histiocytosis is a group of diverse disorders from mutations that lead to an accumulation of immune cells termed histiocytes. Despite the numerous numbers and types of histiocytic diseases, the Histiocyte Society has classified them into five main category groups: L, C, M, R, and H. We present a case where the pathology does not fall under any of the known classification groups. Our patient exhibited clinical features that suggested it could be classified under any of the five known categories. However, opposing characteristics, IHC evidence, and concurrent symptoms did not align with their respective groups. After multidisciplinary investigations, it was determined that our patient presented with atypical histiocytosis, which most favored the clinical presentation consistent with LCH. The diagnosis of LCH was determined due to the spontaneous resolution of the calvarial lesion in the patient’s clinical course, despite the lack of supporting IHC evidence. Our patient’s atypical pathologic findings delineate the need for further study and cataloging of known histiocytic disorders to help future investigations that involve atypical presentations that do not fall under known classifications.
